# Effects of Dietary Chitosan on Growth Performance, Serum Biochemical Indices, Antioxidant Capacity, and Immune Response of Juvenile Tilapia (*Oreochromis niloticus*) under Cadmium Stress

**DOI:** 10.3390/ani14152259

**Published:** 2024-08-03

**Authors:** Qin Zhang, Yi Xie, Jiaqiong Tang, Liuqing Meng, Enhao Huang, Dongsheng Liu, Tong Tong, Yongqiang Liu, Zhongbao Guo

**Affiliations:** 1 Guangxi Key Laboratory for Polysaccharide Materials and Modifications, Guangxi Minzu University, 158 University Road, Nanning 530008, China; zhangqin@gxmzu.edu.cn (Q.Z.); xieyi@stu.gxmzu.edu.cn (Y.X.); tangjiaqiong2024@163.com (J.T.); mengliuqing@stu.gxmzu.edu.cn (L.M.); huangenhao@stu.gxmzu.edu.cn (E.H.); liudongsheng@stu.gxmzu.edu.cn (D.L.); tongtong@gxmzu.edu.cn (T.T.); 2 Guangxi Marine Microbial Resources Industrialization Engineering Technology Research Center, Guangxi Minzu University, 158 University Road, Nanning 530008, China; 3 School of Marine Sciences and Biotechnology, Guangxi Minzu University, 158 University Road, Nanning 530008, China; 4 Guangxi Academy of Fishery Science, 8 Qingshan Road, Nanning 530021, China

**Keywords:** heavy metal, inflammatory response, gene expression, juvenile GIFT

## Abstract

**Simple Summary:**

Cadmium is a toxic non-essential transition metal and poses significant risks to diverse organisms residing in aquatic ecosystems. Cadmium has the potential to build up in various levels of organisms within a food chain, which may pose a risk to human health as it progresses through the trophic levels. The presence of abundant hydroxyl and amino groups in chitosan enables effective adsorption and binding of heavy metal ions, thereby facilitating the removal of diverse heavy metal pollutants and water quality purification. In this study, five groups of juvenile tilapias (initial body weight 21.21 ± 0.24 g) were fed five diets with different levels (0%, 0.5%, 1.0%, 1.5%, and 2.0%) of chitosan supplementation for 60 days under cadmium stress (0.2 mg/L Cd^2+^). The findings suggested that the growth performance, serum biochemical indices, antioxidant capacity, immune response, inflammatory response, and expression of relevant genes in juvenile GIFT could be positively affected by dietary chitosan under cadmium stress.

**Abstract:**

The objective of this study was to examine the effects of varying levels of dietary chitosan supplementation on mitigating cadmium stress and its influence on growth performance, serum biochemical indices, antioxidant capacity, immune response, inflammatory response, and the expression of related genes in juvenile Genetically Improved Farmed Tilapia (GIFT, *Oreochromis niloticus*). Five groups of juvenile tilapias (initial body weight 21.21 ± 0.24 g) were fed five diets with different levels (0%, 0.5%, 1.0%, 1.5%, and 2.0%) of chitosan supplementation for 60 days under cadmium stress (0.2 mg/L Cd^2+^). The findings indicated that, compared with the 0% chitosan group, dietary chitosan could significantly increase (*p* < 0.05) the final weight (Wf), weight gain rate (WGR), specific growth rate (SGR), daily growth index (DGI), and condition factor (CF), while the feed conversion ratio (FCR) expressed the opposite trend in juvenile GIFT. Dietary chitosan could significantly increase (*p* < 0.05) the activities (contents) of cholinesterase (CHE), albumin (ALB), lactate dehydrogenase (LDH), alkaline phosphatase (ALP), acid phosphatase (ACP), and lysozyme (LZM), while glutamic pyruvic transaminase (GPT), glutamic oxaloacetic transaminase (GOT), and complement 3 (C3) in the serum of juvenile GIFT expressed the opposite trend. Dietary chitosan could significantly increase (*p* < 0.05) the activities of superoxide dismutase (SOD) and catalase (CAT) and significantly decrease (*p* < 0.05) the activities (contents) of glutathione S-transferase (GST), glutathione peroxidase (GSH-Px), and malondialdehyde (MDA) in the serum of juvenile GIFT. Dietary chitosan could significantly increase (*p* < 0.05) the activities (contents) of CAT, GST, GSH-Px, and total antioxidant capacity (T-AOC) and significantly decrease (*p* < 0.05) the contents of MDA in the liver of juvenile GIFT. Dietary chitosan could significantly increase (*p* < 0.05) the activities (contents) of SOD, GSH-Px, T-AOC, Na^+^-K^+^-ATPase, and Ca^2+^-ATPase and significantly decrease (*p* < 0.05) the activities (contents) of CAT, GST, and MDA in the gills of juvenile GIFT. Dietary chitosan could significantly up-regulate (*p* < 0.05) the gene expression of *cat*, *sod*, *gst*, and *gsh-px* in the liver of juvenile GIFT. Dietary chitosan could significantly up-regulate (*p* < 0.05) the gene expression of interferon-γ (*inf-γ*) in the gills and spleen and significantly down-regulate (*p* < 0.05) the gene expression of *inf-γ* in the liver and head kidney of juvenile GIFT. Dietary chitosan could significantly down-regulate (*p* < 0.05) the gene expression of interleukin-6 (*il-6*), *il-8*, and tumor necrosis factor-α (*tnf-α*) in the liver, gills, head kidney, and spleen of juvenile GIFT. Dietary chitosan could significantly up-regulate (*p* < 0.05) the gene expression of *il-10* in the liver, gills, head kidney, and spleen of juvenile GIFT. Dietary chitosan could significantly up-regulate (*p* < 0.05) the gene expression of transforming growth factor-β (*tgf-β*) in the liver and significantly down-regulate (*p* < 0.05) the gene expression of *tgf-β* in the head kidney and spleen of juvenile GIFT. In conclusion, dietary chitosan could mitigate the impact of cadmium stress on growth performance, serum biochemical indices, antioxidant capacity, immune response, inflammatory response, and related gene expression in juvenile GIFT. According to the analysis of second-order polynomial regression, it was found that the optimal dietary chitosan levels in juvenile GIFT was approximately 1.42% to 1.45%, based on its impact on Wf, WGR, SGR, and DGI.

## 1. Introduction

Cadmium (Cd) is a toxic non-essential transition metal and poses significant risks to diverse organisms residing in aquatic ecosystems. It is widely acknowledged as a prominent pollutant in water bodies globally. Cadmium can accumulate in organisms at different trophic levels, posing a potential threat to human health as it travels up the food chain [1]. The presence of cadmium in aquatic environments can be attributed to the application and exploitation of fertilizers in agricultural practices, the utilization of metals and minerals in industrial processes, as well as the incorporation of certain industrial products containing cadmium that might be released into water alongside wastewater [2]. Cadmium possesses high levels of environmental toxicity, facilitates easy accumulation, and exhibits limited degradation capabilities. It exerts toxic effects on various organs within organisms. Detecting cadmium contamination is challenging due to its elusive nature, yet it has a propensity for enrichment and can transmit its harmful effects through the food chain [3]. While the concentration of cadmium may be low at times, it can build up in algae and sediment. It can be taken in by aquatic organisms such as fish, shellfish, shrimp, and crabs, which can accumulate cadmium through the food chain, ultimately causing irreversible damage to aquatic tissues and organs, such as inflammation, oxidative stress, and tissue lesions [4,5]. The current methods commonly employed for controlling heavy metal pollution in water bodies include physical dilution, chemical precipitation, ion exchange, and bioremediation. However, these methods are associated with drawbacks such as high costs, potential for secondary pollution, adverse environmental impact, and prolonged treatment duration [6,7,8]. Therefore, finding a treatment method that is biodegradable, environmentally friendly, non-toxic, cost-effective, and efficient has become an urgent priority.

Chitin is abundantly present in crustaceans, insect exoskeletons, and fungal cell walls, and it ranks second only to cellulose as a mucopolysaccharide in nature. Due to its remarkable biological adaptability, non-toxicity, and biodegradability by microorganisms, it has garnered significant attention. However, its limited solubility and chemical inertness have hindered its widespread application [9]. Chitosan, the deacetylation product of chitin, is currently the only alkaline polysaccharide discovered. Due to its superior solubility compared to chitin, it finds extensive applications in the fields of food, medicine, cosmetics, and water treatment [10]. The studies have demonstrated that chitosan possesses bioremediation capabilities, promotes growth, regulates nutrient metabolism, and enhances disease resistance in aquatic animals. Consequently, it can serve as a substitute for conventional chemical drugs and antibiotics, thereby fostering the sustainable development of the aquaculture industry [11]. In addition, the presence of abundant hydroxyl and amino groups in chitosan enables effective chelation of heavy metal ions, thereby facilitating the removal of diverse heavy metal pollutants and improving water quality purification [12].

The fish holds significant ecological value within the aquatic ecosystem and exhibits high susceptibility towards cadmium contamination. Prolonged exposure to cadmium pollution can impede their growth, disrupt physiological metabolism, hinder reproductive capabilities, and compromise immune system functionality, ultimately resulting in metabolic dysfunctions and potential mortality [13,14]. The Genetically Improved Farmed Tilapia (GIFT, *Oreochromis niloticus*) has been genetically enhanced for farming purposes and widely recognized as a highly valuable fish species worldwide. It is considered an excellent choice for experimental studies due to its exceptional growth rate, adaptability to different environments, rich nutritional composition, ease of reproduction, and various other advantageous traits [15]. Many tilapia-farming practices employ intensive farming methods, which can potentially lead to heavy metal pollution. However, there are few studies on adding chitosan to the diet of tilapia to alleviate heavy metal stress. Therefore, the objective of this study was to examine the effects of varying levels of dietary chitosan supplementation on mitigating cadmium stress and its influence on growth performance, serum biochemical indices, antioxidant capacity, immune response, inflammatory response, and the expression of related genes in juvenile GIFT. The findings of this study offer a scientific basis for the utilization of chitosan in the field of aquaculture.

## 2. Materials and Methods

### 2.1. Cadmium and Chitosan

The cadmium chloride (CdCl_2_) Analytical Reagent (AR) utilized in this study was procured from Sigma-Aldrich (Shanghai) Trading Co. Ltd., located in Shanghai, China. The chitosan employed was a food-grade variant, possessing a deacetylation degree of at least 95% and exhibiting viscosity within the range of 100–200 mpa.s. It was procured from Shanghai Yichen Biotechnology Co., LTD., located in Shanghai, China, under the brand name Macklin.

### 2.2. Experimental Diets

In this study, following the findings of Zhang et al. and Liu et al. [1,15], five diets with different levels (0%, 0.5%, 1.0%, 1.5%, and 2.0%) of chitosan supplementation were designed. The composition of the experimental diets for juvenile GIFT can be found in Table 1.

All the feed ingredients were obtained from Nanning Haida Feed Co., LTD., located in Nanning, China, and they met the standards for animal food-grade quality. The process of making different diets in pellet form involved several steps: First, all raw materials were precisely measured according to the specified formula (as outlined in Table 1). Second, all solid components, except for fish oil and soybean oil, were meticulously pulverized and filtered using a 60-mesh sieve. Subsequently, they were mixed thoroughly in blending equipment for 15 min. Third, the mixture was conditioned and matured, then extruded into pellets of 1.0–3.0 mm size using an expanding machine. The size of the pellets was modified according to the fish’s body weight. The pellets underwent dehydration in a hot-air oven until achieving a moisture content lower than 10%. Finally, the pellets were uniformly coated with the remaining fish oil and soybean oil using a vacuum spraying technique. Subsequently, the packaged feed was stored at a temperature of −20 °C in appropriately labeled plastic bags.

### 2.3. Experimental Fish, Acclimatization, and Culture

The experimental juvenile GIFT were acquired from the Seed-breeding Farm of the Guangxi Academy of Fishery Sciences located in Nanning, China. The Ethics Committee of Guangxi Minzu University, Nanning, China, granted permission for utilizing juvenile GIFT in this study with the reference number GXMZU-2022-001.

After being disinfected with a potassium permanganate solution at a concentration of 10 mg/L for 15 min, the juvenile GIFT underwent a 14-day acclimatization phase in an aquaculture system within the laboratory facilities of Guangxi Minzu University. The acclimation conditions included a water temperature of 26 ± 1 °C, pH levels between 7 and 8, dissolved oxygen levels exceeding 8 mg/L, and a light–dark cycle of 12 h each. The juveniles received three meals per day at 9:00, 14:00, and 19:00, following a control diet with no chitosan content. The feeding amounts were modified until the fish exhibited no additional feeding activity during the designated mealtimes.

After a period of 14 days for adaptation, a total of 450 juveniles, initially weighing 21.21 ± 0.24 g, were randomly assigned to five groups, each consisting of three replicates. This resulted in a total of 15 tanks, with each tank accommodating 30 fish for the formal experiment. Each tank had a volume of 360 L (1 m × 0.6 m × 0.6 m). The juvenile GIFT was reared under the same breeding conditions as described during the acclimatization phase. The juveniles were fed for 60 days using one of 5 diets above (Table 1) at a daily rate of 5% of their total wet weight at the feeding time.

Based on previous research findings [1], it was observed that the concentration of Cd^2+^ in water exceeded 0.2 mg/L, leading to a noticeable impact on the growth performance of juvenile GIFT. The juvenile GIFT used in both experiments were the same size. As a result, a constant concentration of Cd^2+^ at 0.2 mg/L was maintained in all 15 tanks containing aquaculture water samples. Throughout the entire duration of the experiment, one-third of the aquaculture water in each tank was replaced daily, accompanied by consistent addition of Cd^2+^ solution with fixed volume and concentration. This process ensured a stable Cd^2+^ concentration of 0.2 mg/L across all tanks over the 60-day experiment duration.

### 2.4. Sampling

After a 60-day experimental period, the juveniles were subjected to a fasting phase lasting for 24 h. Subsequently, a total of 18 juveniles were randomly selected from each experimental group (6 juveniles per tank). These chosen individuals were individually anesthetized using ethyl 3-aminobenzoate mesylate (MS-222) at a concentration of 0.2 g/L, obtained from Adamas Reagent Co., Ltd., Shanghai, China. Their individual body weight and length measurements were then taken to calculate their growth performance. 

The caudal vein of fish was used to collect blood, which was then transferred into a sterile Eppendorf tube. Subsequently, the samples were refrigerated at 4 °C for a duration of 24 h prior to being subjected to centrifugation at 4 °C and 3000× *g* for a period of 15 min. The resulting upper serum layer was carefully extracted, transferred to a new Eppendorf tube, and appropriately labeled. The sample fish was placed on ice and a pair of scissors was utilized to carefully remove one side of the fish’s muscle, exposing its internal organs completely. The liver and gills for antioxidant enzyme analysis and the spleen, head kidney, gills, and liver for gene expression analysis were obtained from each fish and carefully placed in labeled sample bags. These samples were then preserved using liquid nitrogen before being stored at −80 °C in an ultra-low temperature refrigerator for potential future analysis.

### 2.5. Calculation of Growth Performance

The survival rate (SR), weight gain rate (WGR), specific growth rate (SGR), daily growth index (DGI), feed conversion ratio (FCR), condition factor (CF), hepatosomatic index (HSI), viscera index (VSI), and spleen index (SI) of juvenile GIFT were calculated using the following formula:

$$\mathrm{SR}\;(\%)=100\;\times \;\frac{\mathrm{final}\;\mathrm{number}\;\mathrm{of}\;\mathrm{fish}}{\;\mathrm{initial}\;\mathrm{number}\;\mathrm{of}\;\mathrm{fish}}$$

$$\mathrm{WGR}\;(\%)=100\;\times \;\frac{\mathrm{final}\;\mathrm{body}\;\mathrm{weight}\;(g)\;-\;\mathrm{initial}\;\mathrm{body}\;\mathrm{weight}\;(g)}{\mathrm{initial}\;\mathrm{body}\;\mathrm{weight}\;(g)}$$

$$\mathrm{SGR}\;(\%/d)=100\;\times \;\frac{[\ln(\mathrm{final}\;\mathrm{body}\;\mathrm{weight})\;(g)]\;-[\ln\left(\mathrm{initial}\;\mathrm{body}\;\mathrm{weight})\;(g)\right]}{\mathrm{days}}$$

$$\mathrm{DGI}\;(\%/d)=100\;\times \;\frac{[{\left(\mathrm{final}\;\mathrm{body}\;\mathrm{weight})\;(g)\right]}^{1/3}-[{\left(\mathrm{initial}\;\mathrm{body}\;\mathrm{weight})\;(g)\right]}^{1/3}}{\mathrm{days}}$$

$$\mathrm{FCR}=\frac{\mathrm{total}\;\mathrm{feed}\;\mathrm{intake}\;(g)}{\mathrm{final}\;\mathrm{body}\;\mathrm{weight}\;(g)\;-\;\mathrm{initial}\;\mathrm{body}\;\mathrm{weight}\;(g)}$$

$$\mathrm{CF}\;(\%)=100\;\times \;\frac{\mathrm{body}\;\mathrm{weight}\;(g)}{{\left[\mathrm{body}\;\mathrm{length}\;(\mathrm{cm})\right]}^{3}}$$

$$\mathrm{HSI}\;(\%)=100\;\times \;\frac{\mathrm{liver}\;\mathrm{weight}\;(g)\;}{\mathrm{body}\;\mathrm{weight}\;(g)\;}\;\mathrm{VSI}\;(\%)=100\;\times \;\frac{\mathrm{viscera}\;\mathrm{weight}\;(g)\;}{\mathrm{body}\;\mathrm{weight}\;(g)}\;\mathrm{SI}\;(\%)=100\;\times \;\frac{\mathrm{spleen}\;\mathrm{weight}\;(g)\;}{\mathrm{body}\;\mathrm{weight}\;(g)}$$

### 2.6. Determination of Serum Biochemical Indexes, Antioxidant Enzyme Activity, and ATPase Activity

According to the methods of Zhang et al. [16], the cholinesterase (CHE), albumin (ALB), immunoglobulin M (IgM), complement 3 (C3), glutamic pyruvic transaminase (GPT), glutamic oxaloacetic transaminase (GOT), lactate dehydrogenase (LDH), alkaline phosphatase (ALP), acid phosphatase (ACP), and lysozyme (LZM) in the serum; the superoxide dismutase (SOD), malondialdehyde (MDA), glutathione S-transferase (GST), glutathione peroxidase (GSH-Px), total antioxidant capacity (T-AOC), and catalase (CAT) in the serum, gills, and liver; and the Na^+^-K^+^-ATPase and Ca^2+^-ATPase in the gills of juvenile GIFT were determined using an ELISA analyzer (RT-6100, Rayto, Shenzhen, China) and assay kits according to the provided instructions. These kits were manufactured by Nanjing Jiancheng Bioengineering Institute (Nanjing, China), and detailed instructions can be downloaded from http://www.njjcbio.com/ (accessed on 1 May 2024).

### 2.7. Determination of Expression of Antioxidant and Inflammatory Responses Gene

The expression of catalase (*cat*), glutathione peroxidase (*gsh-px*), glutathione S-transferase (*gst*), and superoxide dismutase (*sod*) in the liver and interferon γ (*inf-γ*), interleukin 6 (*il-6*), interleukin 8 (*il-8*), interleukin 10 (*il-10*), tumor necrosis factor α (*tnf-α*), and transforming growth factor β (*tgf-β*) in liver, gills, head kidney, and spleen of juvenile GIFT were analyzed using the real-time quantitative polymerase chain reaction (RT-qPCR) method developed by Zhang et al. [16]. *β-actin* was used as the internal reference gene. The RT-qPCR primers were designed utilizing Primer Premier 6.0 software, with reference to tilapia mRNA sequences obtained from the National Center for Biotechnology Information (NCBI) database. These primers were synthesized by Shanghai Sangon Bioengineering Technology Co., Ltd., located in Shanghai, China. The specific details of these primers can be found in Table 2.

The brief steps of the RT-qPCR method are as follows: First, the liver, gills, head kidney, and spleen of juvenile GIFT were used to extract total RNA. The extraction was performed using the Takara MiniBEST Universal RNA Extraction Kit (Universal type) from Takara Biomedical Technology (Beijing) Co., Ltd. in Beijing, China. Additional guidance for the extraction process was obtained from the kit instructions provided. Subsequently, the RNA’s quantity and purity were assessed using a NanoDrop-2000 spectrophotometer (Thermo, Waltham, MA, USA). To assess the quality of RNA, gel electrophoresis was performed using a 1% (*w*/*v*) agarose TAE gel and stained with Gel Red^TM^ nucleic acid dye (UVP, Upland, CA, USA). Second, the PrimeScript^TM^ RT Master Mix (Perfect Real Time) reverse transcription kit manufactured by Takara Biomedical Technology (Beijing) Co., Ltd. in Beijing, China, was utilized to convert 1000 ng of total RNA into cDNA. The reaction mixture was prepared following the instructions provided with the kit for additional guidance. The reverse transcription PCR reaction was conducted through a single cycle, consisting of incubation at 42 °C for 15 min, followed by heating at 95 °C for 5 min and cooling at 4 °C for 5 s. the RT-qPCR experiments were conducted using a LightCycler 96 RT-qPCR Detection System manufactured by Roche in Basel, Switzerland. Additionally, the TB Green Premix Ex TaqTM II (Tli RNaseH Plus) RT-qPCR kit provided by Takara Biomedical Technology (Beijing) Co., Ltd. in Beijing, China was utilized. The instructions included with the kit offered further guidance for preparing the RT-qPCR reaction mixture. Each sample was tested 3 times. The RT-qPCR reaction commenced with an initial denaturation step at a temperature of 95 °C for a duration of 10 s. This was followed by a series of 40 cycles, involving denaturation at the same temperature (95 °C) for 60 s, annealing at 60 °C for 30 s, and extension at 72 °C for a period of 90 s. To ensure the specificity of the reactions, we verified it by analyzing the melting curve obtained during heating to a temperature of 95 °C.

The expression levels of *cat*, *gsh-px*, *gst*, *sod*, *inf-γ*, *il-6*, *il-8*, *il-10*, *tnf-α*, and *tgf-β* genes in juvenile GIFT were determined using the 2^−∆∆CT^ method [17].

### 2.8. Data Statistical Analyses

The data were initially processed using Microsoft Excel 2023 (Version 16.78 [24060916], Microsoft Corporation, Washington, WA, USA). Subsequently, a one-way analysis of variance (ANOVA) was performed with IBM SPSS 26 software (International Business Machines Corporation, Armonk, NY, USA). Prior to conducting the statistical tests, assessments were made regarding the normality and homogeneity of variances across groups. Group comparisons were carried out utilizing Duncan’s multiple-range test. Statistical significance was determined at a level below *p* < 0.05 and results were presented as mean ± standard error (mean ± SE). To determine the optimal dietary chitosan level for juvenile GIFT in the study, the second-order polynomial regression model proposed was applied by Liu et al. [18].

## 3. Results

### 3.1. Growth Performance

Compared to the 0% chitosan group, the 0.5%, 1.0%, 1.5%, and 2.0% chitosan groups resulted in a significant increase (*p* < 0.05) in final weight (Wf), weight gain rate (WGR), specific growth rate (SGR), daily growth index (DGI), and condition factor (CF) of juvenile GIFT under cadmium stress. The 1.0% chitosan group exhibited the highest values for Wf, WGR, SGR, DGI, and CF, and there were no significant differences (*p* > 0.05) compared to the 0.5%, 1.5%, and 2.0% chitosan groups, respectively, as shown in Table 3.

Compared to the 0% chitosan group, the 1.0%, 1.5%, and 2.0% chitosan groups resulted in a significant increase (*p* < 0.05) in survival rate (SR) of the juvenile GIFT under cadmium stress. However, there is no significant difference (*p* > 0.05) observed between the 0% chitosan group and the 0.5% chitosan group in SR, as shown in Table 3.

Compared to the 0% chitosan group, the 0.5%, 1.0%, 1.5%, and 2.0% chitosan groups resulted in a significant decrease (*p* < 0.05) in feed conversion ratio (FCR) of the juvenile GIFT under cadmium stress. The 1.5% and 2.0% chitosan groups exhibited the lowest values for FCR, which were significantly lower (*p* < 0.05) than that in the 0.5% and 1.0% chitosan groups, as shown in Table 3.

There were no statistically significant differences (*p* > 0.05) observed in the viscera index (VSI), hepatosomatic index (HSI), and spleen index (SI) of juvenile GIFT under cadmium stress when comparing the 0.5%, 1.0%, 1.5%, and 2.0% chitosan groups with the 0% chitosan group, as shown in Table 3.

**Figure 1:**
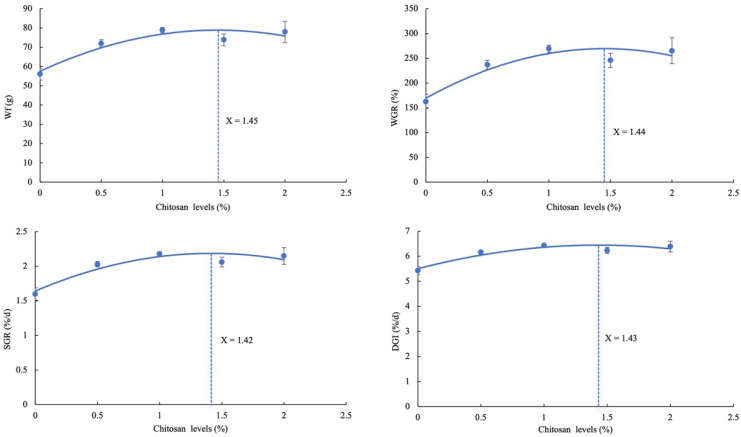
Relationship between different dietary chitosan levels and the final weight (Wf), weight gain rate (WGR), specific growth rate (SGR), and daily growth index (DGI) of juvenile GIFT under cadmium stress based on second-order polynomial regression analysis.

The optimal level of dietary chitosan for Wf in juvenile GIFT exposed to cadmium stress was determined to be 1.45%. This conclusion was derived from a second-order polynomial regression analysis, represented by the equation Y = −10.143X^2^ + 29.426X + 57.547, R^2^ = 0.8784, as shown in Figure 1.

The optimal level of dietary chitosan for the WGR in juvenile GIFT exposed to cadmium stress was determined to be 1.44%. This conclusion was derived from a second-order polynomial regression analysis, represented by the equation Y = −47.486X^2^ + 137.78X + 169.43, R^2^ = 0.8784, as shown in Figure 1.

The optimal level of dietary chitosan for the SGR in juvenile GIFT exposed to cadmium stress was determined to be 1.42%. This conclusion was derived from a second-order polynomial regression analysis, represented by the equation Y = −0.2714X^2^ + 0.7689X + 1.6423, R^2^ = 0.8761, as shown in Figure 1.

The optimal level of dietary chitosan for the DGI in juvenile GIFT exposed to cadmium stress was determined to be 1.43%. This conclusion was derived from a second-order polynomial regression analysis, represented by the equation Y = −0.46X^2^ + 1.318X + 5.5, R^2^ = 0.8826, as shown in Figure 1.

### 3.2. Serum Biochemical Indexes

Compared to the 0% chitosan group, the 0.5%, 1.0%, 1.5%, and 2.0% chitosan groups resulted in a significant increase (*p* < 0.05) in cholinesterase (CHE), albumin (ALB), lactate dehydrogenase (LDH), alkaline phosphatase (ALP), acid phosphatase (ACP), and lysozyme (LZM) in the serum of juvenile GIFT under cadmium stress. The 2.0% chitosan group exhibited the highest value for CHE, which was significantly higher (*p* < 0.05) than that in the 0.5%, 1.0%, and 1.5% chitosan groups. The 2.0% chitosan group exhibited the highest value for ALB, which was significantly higher (*p* < 0.05) than that in the 1.0% chitosan group, but there was no significant difference (*p* > 0.05) compared to the 0.5% and 1.5% chitosan groups. The 1.5% chitosan group exhibited the highest value for LDH, which was significantly higher (*p* < 0.05) than that in the 0.5% and 1.0% chitosan groups, but there was no significant difference (*p* > 0.05) compared to the 2.0% chitosan group. The 2.0% chitosan group exhibited the highest value for ALP, which was significantly higher (*p* < 0.05) than that in the 0.5% and 1.0% chitosan groups, but there was no significant difference (*p* > 0.05) compared to the 1.5% chitosan group. The 1.5% chitosan group exhibited the highest value for ACP, which was significantly higher (*p* < 0.05) than that in the 0.5% chitosan group, but there was no significant difference (*p* > 0.05) compared to the 1.5% and 2.0% chitosan groups. The 2.0% chitosan group exhibited the highest value for LZM, which was significantly higher (*p* < 0.05) than that in the 0.5% chitosan group, but there was no significant difference (*p* > 0.05) compared to the 1.0% and 1.5% chitosan groups, as shown in Table 4.

Compared to the 0% chitosan group, the 0.5%, 1.0%, 1.5%, and 2.0% chitosan groups resulted in a significant decrease (*p* < 0.05) in glutamic pyruvic transaminase (GPT), glutamic oxaloacetic transaminase (GOT), and complement 3 (C3) in the serum of juvenile GIFT under cadmium stress. The 2.0% chitosan group exhibited the lowest value for GPT, which was significantly lower (*p* < 0.05) than that in the 0.5% and 1.0% chitosan groups, but there was no significant difference (*p* > 0.05) compared to the 1.5% chitosan group. The 2.0% chitosan group exhibited the lowest value for GOT, which was significantly lower (*p* < 0.05) than that in the 0.5%, 1.0%, and 1.5% chitosan groups. The 2.0% chitosan group exhibited the lowest value for C3, which was significantly lower (*p* < 0.05) than that in the 0.5% chitosan group, but there was no significant difference (*p* > 0.05) compared to the 1.0% and 1.5% chitosan group, as shown in Table 4.

Compared to the 0% chitosan group, the 1.0%, 1.5%, and 2.0% chitosan groups resulted in a significant decrease (*p* < 0.05) in immunoglobulin M (IgM) in the serum of juvenile GIFT under cadmium stress. The 2.0% chitosan group exhibited the lowest value for IgM, which was significantly lower (*p* < 0.05) than that in the 1.0% and 1.5% chitosan groups. However, there was no statistically significant difference (*p* > 0.05) observed in the IgM of juvenile GIFT under cadmium stress when comparing the 0.5% chitosan group with the 0% chitosan group, as shown in Table 4.

### 3.3. Antioxidant Enzyme Activity

Compared to the 0% chitosan group, the 0.5%, 1.0%, 1.5%, and 2.0% chitosan groups resulted in a significant increase (*p* < 0.05) in superoxide dismutase (SOD) and catalase (CAT) in the serum of juvenile GIFT under cadmium stress. The 1.5% chitosan group exhibited the highest value for SOD, which was significantly higher (*p* < 0.05) than that in the 0.5%, 1.0%, and 2.0% chitosan groups. The 2.0% chitosan group exhibited the highest value for CAT, which was significantly higher (*p* < 0.05) than that in the 0.5% and 1.0% chitosan groups, but there was no significant difference (*p* > 0.05) compared to the 1.5% chitosan groups, as shown in Table 5.

Compared to the 0% chitosan group, the 0.5%, 1.0%, 1.5%, and 2.0% chitosan groups resulted in a significant decrease (*p* < 0.05) in glutathione S-transferase (GST), glutathione peroxidase (GSH-Px), and malondialdehyde (MDA) in the serum of juvenile GIFT under cadmium stress. The 2.0% chitosan group exhibited the lowest value for GST and MDA, which were significantly lower (*p* < 0.05) than those in the 0.5%, 1.0%, and 1.5% chitosan groups. The 2.0% chitosan group exhibited the lowest value for GSH-Px, which was significantly lower (*p* < 0.05) than that in the 0.5% and 1.0% chitosan groups, but there was no significant difference (*p* > 0.05) compared to the 1.5% chitosan group, as shown in Table 5.

Compared to the 0% chitosan group, the 1.0%, 1.5%, and 2.0% chitosan groups resulted in a significant decrease (*p* < 0.05) in total antioxidant capacity (T-AOC) in the serum of juvenile GIFT under cadmium stress. The 2.0% chitosan group exhibited the lowest value for T-AOC, which was significantly lower (*p* < 0.05) than that in the 1.0% chitosan group, but there was no significant difference (*p* > 0.05) compared to the 1.5% chitosan group. However, there was no statistically significant difference (*p* > 0.05) observed in the T-AOC of juvenile GIFT under cadmium stress when comparing the 0.5% chitosan group with the 0% chitosan group, as shown in Table 5. However, there was no significant difference (*p* > 0.05) between the 0.5% group and control group. The 2.0% group exhibited the lowest T-AOC, which was significantly lower (*p* < 0.05) than that in the 1.0% group, but there was no significant difference (*p* > 0.05) compared to the 1.5% group, as shown in Table 5.

Compared to the 0% chitosan group, the 0.5%, 1.0%, 1.5%, and 2.0% chitosan groups resulted in a significant increase (*p* < 0.05) in SOD, CAT, GSH-Px, and T-AOC in the liver of juvenile GIFT under cadmium stress. The 2.0% chitosan group exhibited the highest value for SOD, which was significantly higher (*p* < 0.05) than that in the 0.5% chitosan group, but there was no significant difference (*p* > 0.05) compared to the 1.0% and 1.5% chitosan groups. The 1.5% chitosan group exhibited the highest value for CAT, which was significantly higher (*p* < 0.05) than that in the 0.5%, 1.0%, and 2.0% chitosan groups. The 0.5% chitosan group exhibited the highest value for GSH-Px, but there was no significant difference (*p* > 0.05) compared to the 1.0%, 1.5%, and 2.0% chitosan groups. The 2.0% chitosan group exhibited the highest value for T-AOC, which was significantly higher (*p* < 0.05) than that in the 0.5% and 1.0% chitosan groups, but there was no significant difference (*p* > 0.05) compared to the 1.5% chitosan group, as shown in Table 6. 

Compared to the 0% chitosan group, the 0.5%, 1.0%, 1.5%, and 2.0% chitosan groups resulted in a significant increase (*p* < 0.05) in GST in the liver of juvenile GIFT under cadmium stress. The 2.0% chitosan group exhibited the highest value for GST, which was significantly higher (*p* < 0.05) than that in the 1.0% chitosan group, but there was no significant difference (*p* > 0.05) compared to the 1.5% chitosan group. However, there was no statistically significant difference (*p* > 0.05) observed in the GST of juvenile GIFT under cadmium stress when comparing the 0.5% chitosan group with the 0% chitosan group, as shown in Table 6.

Compared to the 0% chitosan group, the 0.5%, 1.0%, 1.5%, and 2.0% chitosan groups resulted in a significant decrease (*p* < 0.05) in MDA in the liver of juvenile GIFT under cadmium stress. The 2.0% chitosan group exhibited the lowest value for MDA, which was significantly lower (*p* < 0.05) than that in the 0.5%, 1.0%, and 1.5% chitosan groups, as shown in Table 6.

Compared to the 0% chitosan group, the 0.5%, 1.0%, 1.5%, and 2.0% chitosan groups resulted in a significant increase (*p* < 0.05) in SOD, GSH-Px, and T-AOC in the gills of juvenile GIFT under cadmium stress. The 1.5% chitosan group exhibited the highest value for SOD, which was significantly higher (*p* < 0.05) than that in the 0.5%, 1.0%, and 2.0% chitosan groups. The 1.5% chitosan group exhibited the highest value for GSH-Px, which was significantly higher (*p* < 0.05) than that in the 0.5% chitosan group, but there was no significant difference (*p* > 0.05) compared to the 1.0% and 1.5% chitosan groups. The 2.0% chitosan group exhibited the highest value for T-AOC, but there was no significant difference (*p* > 0.05) compared to the 0.5%, 1.0%, and 1.5% chitosan groups, as shown in Table 7.

Compared to the 0% chitosan group, the 0.5%, 1.0%, 1.5%, and 2.0% chitosan groups resulted in a significant decrease (*p* < 0.05) in CAT, GST, and MDA in the gills of juvenile GIFT under cadmium stress. The 2.0% chitosan group exhibited the lowest value for CAT, but there was no significant difference (*p* > 0.05) compared to the 0.5%, 1.0%, and 1.5% chitosan groups. The 2.0% chitosan group exhibited the lowest value for GST, which was significantly lower (*p* < 0.05) than that in the 0.5% and 1.0% chitosan groups, but there was no significant difference (*p* > 0.05) compared to the 1.5% chitosan group. The 2.0% chitosan group exhibited the lowest value for MDA, which was significantly lower (*p* < 0.05) than that in the 0.5% chitosan group, but there was no significant difference (*p* > 0.05) compared to the 1.0% and 1.5% chitosan groups, as shown in Table 7.

### 3.4. ATPase Activity

**Figure 2:**
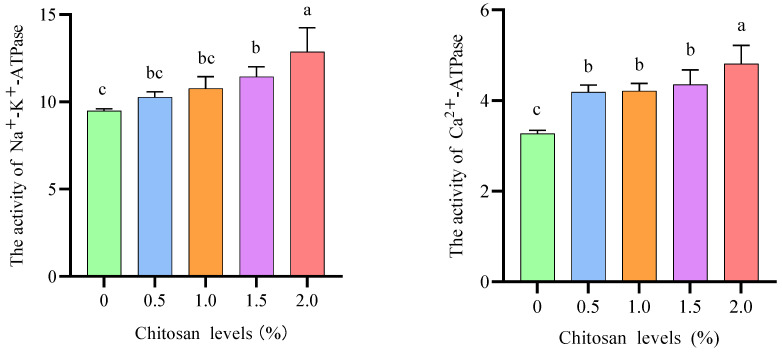
Effects of dietary chitosan on ATPase activity in the gills of juvenile GIFT under cadmium stress. All the above data are mean ± SE (*n* = 3). Different superscript letters in the figure indicate significant differences among the data (*p* < 0.05).

Compared to the 0% chitosan group, the 1.5% and 2.0% chitosan groups resulted in a significant increase (*p* < 0.05) in the Na^+^-K^+^-ATPase in the gills of juvenile GIFT under cadmium stress. The 2.0% chitosan group exhibited the highest value for Na^+^-K^+^-ATPase, which was significantly higher (*p* < 0.05) than that in the 1.5% chitosan group. However, there was no statistically significant difference (*p* > 0.05) observed in the Na^+^-K^+^-ATPase of juvenile GIFT under cadmium stress when comparing the 0.5% and 1.0% chitosan groups with the 0% chitosan group, as shown in Figure 2.

Compared to the 0% chitosan group, the 0.5%, 1.0%, 1.5%, and 2.0% chitosan groups resulted in a significant increase (*p* < 0.05) in the Ca^2+^-ATPase in the gills of juvenile GIFT under cadmium stress. The 2.0% chitosan group exhibited the highest value for Ca^2+^-ATPase, which was significantly higher (*p* < 0.05) than that in the 0.5%, 1.0%, and 1.5% chitosan groups, as shown in Figure 2.

### 3.5. Relative Expression of the Antioxidant Gene

**Figure 3:**
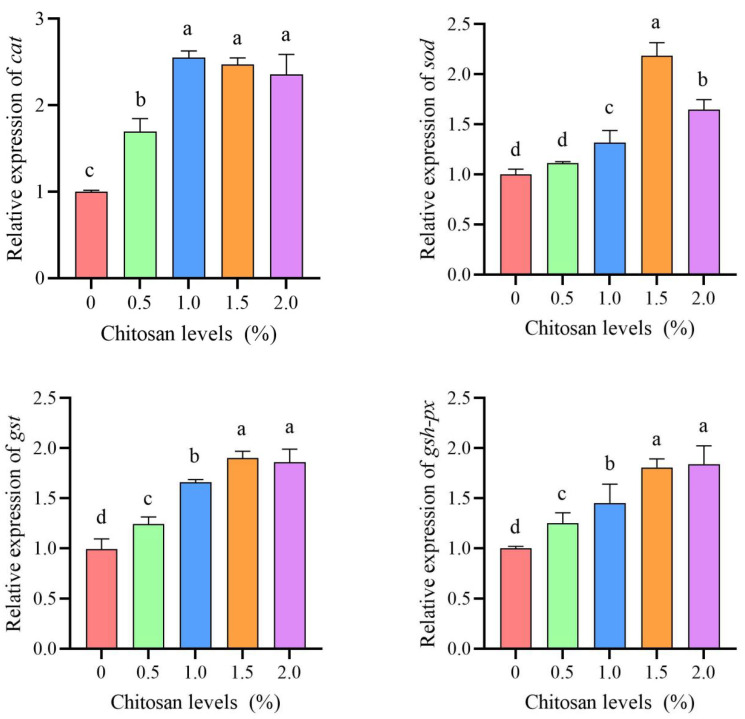
Effects of dietary chitosan on the relative expression of catalase (*cat*), superoxide dismutase (*sod*), glutathione S-transferase (*gst*), and glutathione peroxidase (*gsh-px*) in the liver of juvenile GIFT under cadmium stress. All the above data are mean ± SE (*n* = 3). Different superscript letters in the figure indicate significant differences among the data (*p* < 0.05).

Compared to the 0% chitosan group, the 0.5%, 1.0%, 1.5%, and 2.0% chitosan groups resulted in a significant increase (*p* < 0.05) in the relative expression of *cat*, *gst*, and *gsh-px* in the liver of juvenile GIFT under cadmium stress. The 1.0% chitosan group exhibited the highest value for *cat*, which was significantly higher (*p* < 0.05) than that in the 0.5% chitosan group, but there was no significant difference (*p* > 0.05) compared to the 1.5% and 2.0% chitosan groups. The 1.5% chitosan group exhibited the highest value for *gst*, which was significantly higher (*p* < 0.05) than that in the 0.5% and 1.0% chitosan groups, but there was no significant difference (*p* > 0.05) compared to the 2.0% chitosan group. The 2.0% chitosan group exhibited the highest value for *gsh-px*, which was significantly higher (*p* < 0.05) than that in the 0.5% and 1.0% chitosan groups, but there was no significant difference (*p* > 0.05) compared to the 1.5% chitosan group, as shown in Figure 3.

Compared to the 0% chitosan group, the 1.0%, 1.5%, and 2.0% chitosan groups resulted in a significant increase (*p* < 0.05) in the relative expression of *sod* in the liver of juvenile GIFT under cadmium stress. The 1.5% chitosan group exhibited the highest value for *sod*, which was significantly higher (*p* < 0.05) than that in the 1.0% and 2.0% chitosan groups, but there was no significant difference (*p* > 0.05) compared to the 1.5% and 2.0% chitosan groups. However, there was no statistically significant difference (*p* > 0.05) observed in the *sod* of juvenile GIFT under cadmium stress when comparing the 0.5% chitosan group with the 0% chitosan group, as shown in Figure 3.

### 3.6. Relative Expression of Inflammatory Response Gene

**Figure 4:**
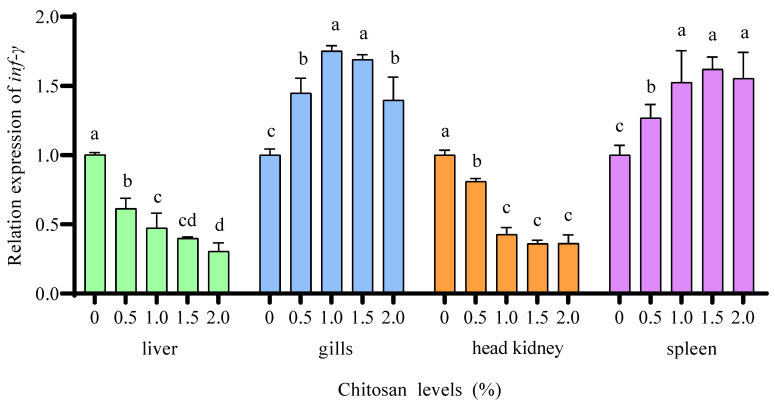
Effects of dietary chitosan on the relative expression of interferon-γ (*inf-γ*) in the liver, gills, head kidney, and spleen of juvenile GIFT under cadmium stress. All the above data are mean ± SE (*n* = 3). Different superscript letters in the figure indicate significant differences among the data (*p* < 0.05).

Compared to the 0% chitosan group, the 0.5%, 1.0%, 1.5%, and 2.0% chitosan groups resulted in a significant increase (*p* < 0.05) in the relative expression of *inf-γ* in the gills and spleen of juvenile GIFT under cadmium stress. The 1.0% chitosan group exhibited the highest value for *inf-γ* in the gills, which was significantly higher (*p* < 0.05) than that in the 0.5% and 2.0% chitosan groups, but there was no significant difference (*p* > 0.05) compared to the 1.5% chitosan group. The 1.5% chitosan group exhibited the highest value for *inf-γ* in the spleen, which was significantly higher (*p* < 0.05) than that in the 0.5% chitosan group, but there was no significant difference (*p* > 0.05) compared to the 1.0% and 2.0% chitosan groups, as shown in Figure 4.

Compared to the 0% chitosan group, the 0.5%, 1.0%, 1.5%, and 2.0% chitosan groups resulted in a significant decrease (*p* < 0.05) in the relative expression of *inf-γ* in the liver and head kidney of juvenile GIFT under cadmium stress. The 2.0% chitosan group exhibited the lowest value for *inf-γ* in the liver, which was significantly lower (*p* < 0.05) than that in the 0.5% and 1.0% chitosan groups, but there was no significant difference (*p* > 0.05) compared to the 1.5% chitosan group. The 2.0% chitosan group exhibited the lowest value for *inf-γ* in the head kidney, which was significantly lower (*p* < 0.05) than that in the 0.5% chitosan group, but there was no significant difference (*p* > 0.05) compared to the 1.0% and 1.5% chitosan groups, as shown in Figure 4.

**Figure 5:**
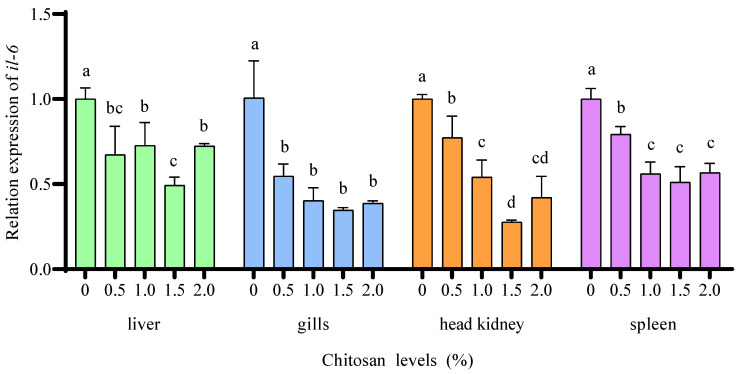
Effects of dietary chitosan on the relative expression of interleukin-6 (*il-6*) in the liver, gills, head kidney, and spleen of juvenile GIFT under cadmium stress. All the above data are mean ± SE (*n* = 3). Different superscript letters in the figure indicate significant differences among the data (*p* < 0.05).

Compared to the 0% chitosan group, the 0.5%, 1.0%, 1.5%, and 2.0% chitosan groups resulted in a significant decreased (*p* < 0.05) the relative expression of *il-6* in the liver, gills, head kidney, and spleen of juvenile GIFT under cadmium stress. The 2.0% chitosan group exhibited the lowest value for *il-6* in the liver, which was significantly lower (*p* < 0.05) than that in the 0.5%, 1.0%, and 2.0% chitosan groups. The 1.5% chitosan group exhibited the lowest value for *il-6* in the gills, but there was no significant difference (*p* > 0.05) compared to the 0.5%, 1.0%, and 2.0% chitosan groups. The 1.5% chitosan group exhibited the lowest value for *il-6* in the head kidney and spleen, which was significantly lower (*p* < 0.05) than that in the 0.5% and 1.0% chitosan groups, but there was no significant difference (*p* > 0.05) compared to the 2.0% chitosan group, as shown in Figure 5.

**Figure 6:**
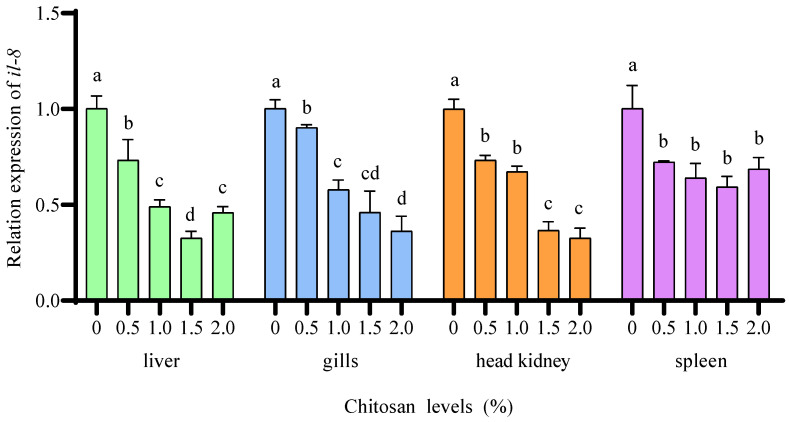
Effects of dietary chitosan on the relative expression of interleukin-8 (*il-8*) in the liver, gills, head kidney, and spleen of juvenile GIFT under cadmium stress. All the above data are mean ± SE (*n* = 3). Different superscript letters in the figure indicate significant differences among the data (*p* < 0.05).

Compared to the 0% chitosan group, the 0.5%, 1.0%, 1.5%, and 2.0% chitosan groups resulted in a significant decrease (*p* < 0.05) in the relative expression of *il-8* in the liver, gills, head kidney, and spleen of juvenile GIFT under cadmium stress. The 1.5% chitosan group exhibited the lowest value for *il-8* in the liver, which was significantly lower (*p* < 0.05) than that in the 0.5%, 1.0%, and 2.0% chitosan groups. The 2.0% chitosan group exhibited the lowest value for *il-8* in the gills and head kidney, which was significantly lower (*p* < 0.05) than that in the 0.5% and 1.0% chitosan groups, but there was no significant difference (*p* > 0.05) compared to the 1.5% chitosan group. The 2.0% chitosan group exhibited the lowest value for *il-8* in the spleen, but there was no significant difference (*p* > 0.05) compared to the 0.5%, 1.0%, and 2.0% chitosan groups, as shown in Figure 6.

**Figure 7:**
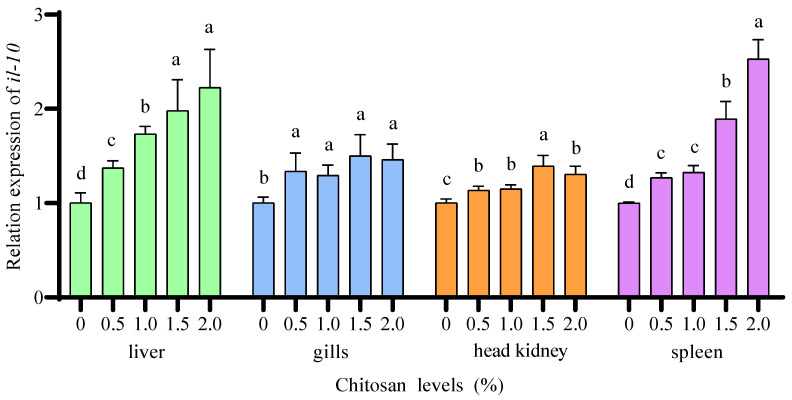
Effects of dietary chitosan on the relative expression of interleukin-10 (*il-10*) in the liver, gills, head kidney, and spleen of juvenile GIFT under cadmium stress. All the above data are mean ± SE (*n* = 3). Different superscript letters in the figure indicate significant differences among the data (*p* < 0.05).

Compared to the 0% chitosan group, the 0.5%, 1.0%, 1.5%, and 2.0% chitosan groups resulted in a significant increase (*p* < 0.05) in the relative expression of *il-10* in the gills, head kidney, and spleen of juvenile GIFT under cadmium stress. The 1.5% chitosan group exhibited the highest value for *il-10* in the gills, but there was no significant difference (*p* > 0.05) compared to the 0.5%, 1.0%, and 2.0% chitosan group. The 1.5% chitosan group exhibited the highest value for *il-10* in the head kidney, which was significantly higher (*p* < 0.05) than that in the 0.5%, 1.0%, and 2.0% chitosan groups. The 2.0% chitosan group exhibited the highest value for *il-10* in the spleen, which was significantly higher (*p* < 0.05) than that in the 0.5%, 1.0%, and 1.5% chitosan groups, as shown in Figure 7.

Compared to the 0% chitosan group, the 1.0%, 1.5%, and 2.0% chitosan groups resulted in a significant increase (*p* < 0.05) in the relative expression of *il-10* in the liver of juvenile GIFT under cadmium stress. The 2.0% chitosan group exhibited the highest value for *il-10* in the liver, which was significantly higher (*p* < 0.05) than that in the 1.0% chitosan group, but there was no significant difference (*p* > 0.05) compared to the 1.5% chitosan group. However, there was no statistically significant difference (*p* > 0.05) observed in the *il-10* of juvenile GIFT under cadmium stress when comparing the 0.5% chitosan group with the 0% chitosan group, as shown in Figure 7.

**Figure 8:**
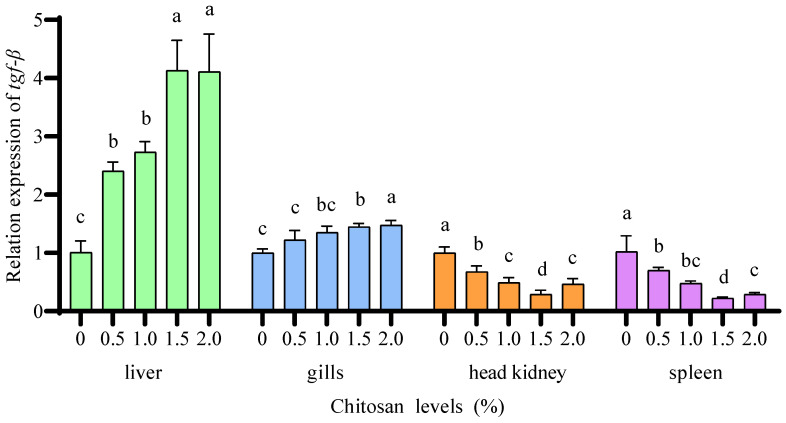
Effects of dietary chitosan on the relative expression of transforming growth factor-β (*tgf-β*) in the liver, gills, head kidney, and spleen of juvenile GIFT under cadmium stress. All the above data are mean ± SE (*n* = 3). Different superscript letters in the figure indicate significant differences among the data (*p* < 0.05).

Compared to the 0% chitosan group, the 0.5%, 1.0%, 1.5%, and 2.0% chitosan groups resulted in a significant increase (*p* < 0.05) in the relative expression of *tgf-β* in the liver of juvenile GIFT under cadmium stress. The 2.0% chitosan group exhibited the highest value for *tgf-β* in the liver, which was significantly higher (*p* < 0.05) than that in the 0.5% and 1.0% chitosan groups, but there was no significant difference (*p* > 0.05) compared to the 1.5% chitosan group, as shown in Figure 8.

Compared to the 0% chitosan group, the 1.0%, 1.5%, and 2.0% chitosan groups resulted in a significant increase (*p* < 0.05) in the relative expression of *tgf-β* in the gills of juvenile GIFT under cadmium stress. The 2.0% chitosan group exhibited the highest value for *tgf-β* in the gills, which was significantly higher (*p* < 0.05) than that in the 0.5% and 1.0% chitosan groups, However, there was no statistically significant difference (*p* > 0.05) observed in the *tgf-β* of juvenile GIFT under cadmium stress when comparing the 0.5% chitosan group with the 0% chitosan group, as shown in Figure 8.

Compared to the 0% chitosan group, the 0.5%, 1.0%, 1.5%, and 2.0% chitosan groups resulted in a significant decrease (*p* < 0.05) in the relative expression of *tgf-β* in the head kidney and spleen of juvenile GIFT under cadmium stress. The 1.5% chitosan group exhibited the lowest value for *tgf-β* in the head kidney and spleen, which was significantly lower (*p* < 0.05) than that in the 0.5%, 1.0%, and 2.0% chitosan groups, as shown in Figure 8.

**Figure 9:**
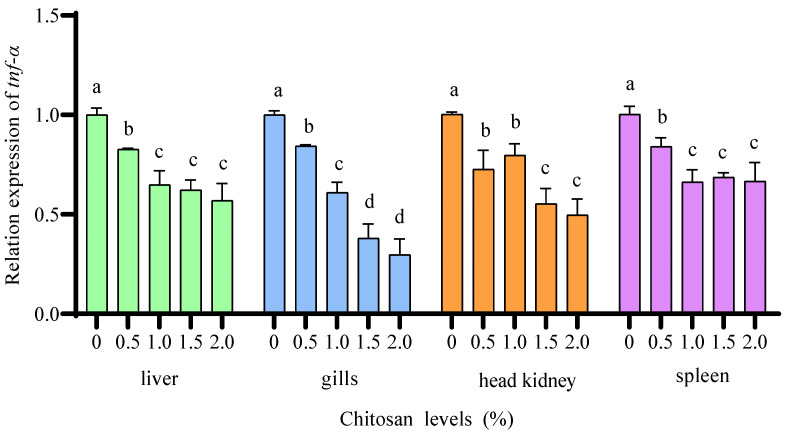
Effects of dietary chitosan on the relative expression of tumor necrosis factor-α (*tnf-α*) in the liver, gills, head kidney, and spleen of juvenile GIFT under cadmium stress. All the above data are mean ± SE (*n* = 3). Different superscript letters in the figure indicate significant differences among the data (*p* < 0.05).

Compared to the 0% chitosan group, the 0.5%, 1.0%, 1.5%, and 2.0% chitosan groups resulted in a significant decrease (*p* < 0.05) in the relative expression of *tnf-α* in the liver, gills, head kidney, and spleen of juvenile GIFT under cadmium stress. The 2.0% chitosan group exhibited the lowest value for *tnf-α* in the liver and spleen, which was significantly lower (*p* < 0.05) than that in the 0.5% chitosan group, but there was no significant difference (*p* > 0.05) compared to the 1.0% and 1.5% chitosan groups. The 2.0% chitosan group exhibited the lowest value for *tnf-α* in the gills and head kidney, which was significantly lower (*p* < 0.05) than that in the 0.5% and 1.0% chitosan groups, but there was no significant difference (*p* > 0.05) compared to the 1.5% chitosan group, as shown in Figure 9.

## 4. Discussion

In this study, according to the analysis of the quadratic polynomial regression, the optimal levels of dietary chitosan, in addition to alleviating cadmium stress, were determined to be 1.42–1.45%. However, exceeding the optimal levels showed no positive impact of chitosan on the growth of juvenile GIFT. Optimal dietary chitosan levels could significantly improve the growth performance of juvenile GIFT and alleviate the inhibitory effect of cadmium stress on growth performance. Similar studies are as follows: The study of Wu showed that the diets containing 0.4% chitosan increased WGR, FCR, and SGR of tilapia, and the findings provide evidence supporting the growth-promoting effects of chitosan on tilapia. [19]. The study of Chen et al. showed that diets containing 1 or 5 g/kg chitosan significantly increased the WGR, SGR, CF, and SR of loaches (*Misgurnus anguillicadatus*), and these results indicated that chitosan could promote growth in loaches [20]. This may be because chitosan could form a chelating polymer with heavy metals, which alleviated the inhibition of energy metabolism caused by heavy metals in fish and reduced the accumulation of heavy metals in fish. As a result, it slowed down the inhibitory effect of heavy metals on fish growth [21]. In addition, to attribute the beneficial effect of chitosan on growth performance might increase absorption and assimilation of nutrients at a lower concentration in freshwater fish. Chitosan could enhance the appetite of aquatic animals, boost the activity of intestinal digestive enzymes, augment the thickness of intestinal mucosal folds, and expand the absorption area, thereby enhancing nutrient digestion and absorption [1,22]. El-Naby et al. reported the enhancement of digestive enzyme (lipase and amylase) activity following the addition of chitosan to Nile tilapia diets [23]. 

Serum biochemical indexes of fish are closely related to metabolism, nutrient absorption, and health status. They are important indexes to evaluate physiology and pathology and are widely used to measure metabolism and health status [24]. The serum albumin (ALB) content serves as an indicator of the body’s metabolic and protein absorption status while also directly reflecting its non-specific immune function [25]. This study demonstrate that dietary chitosan significantly enhances the content of ALB in the serum, thereby indicating its potential to promote metabolism and protein absorption, as well as improve non-specific immune function. Lysozyme (LZM) is one of the non-specific immune factors present in fish blood, and its activity serves as an indicator of bacteriolytic action strength, determining whether phagocytes can effectively eliminate phagocytic pathogens [26]. Alkaline phosphatase (ALP) and acid phosphatase (ACP) play a direct role in phosphate group transfer and metabolism in fish, which is closely associated with protein and lipid metabolism. Additionally, ALP and ACP levels are influenced by nutritional status, environmental changes, diseases, and growth stages [27]. This study demonstrates that dietary chitosan significantly enhanced the activities of ACP and ALP in the serum, thereby indicating chitosan could improve the non-specific immune response of juvenile tilapia. Lactate dehydrogenase (LDH), glutamic oxaloacetic transaminase (GOT), and glutamic pyruvic aminotransferase (GPT) AKP are the important indicators of fish physiological activity and disease diagnosis, which can reflect the anti-stress ability of biological organisms [28]. This study demonstrates that dietary chitosan significantly decreased the activities of GOT and GPT and significantly decreased the activities of LDH in the serum, thereby indicating chitosan had a protective effect on liver and could improve the stress resistance of fish. This may be due to the change in protein metabolism under cadmium stress, which induced the change in serum biochemical indexes [29]. The combination of chitosan and cadmium resulted in the formation of a chelating polymer, which exerted cell protection through the generation of amine functional groups upon reaction with metal ions. As a result, it positively influenced serum biochemical indexes [30]. Studies have shown that exposure to toxic metals could decrease the activities of ALP and ACP and increase the activities of GOT and GPT in serum [31,32,33], while dietary chitosan could increase the activities of ALP, ACP, and LDH and decrease the activities of GOT and GPT in serum [34,35,36]. The present experiment involved the addition of chitosan to the diet under cadmium stress, and the obtained experimental results were consistent with those observed when chitosan was incorporated into the diet. This suggests that chitosan can effectively mitigate the inhibitory effects of cadmium stress on fish.

Complement 3 (C3) plays a crucial role in the innate immune response of fish, as it possesses the ability to eliminate pathogens, stimulate inflammatory reactions, and regulate homeostatic cell clearance [37]. Immunoglobulin M (IgM) serves as a pivotal component in the adaptive humoral immunity of bony fish by effectively preventing bacteremia and neutralizing both toxins and viral toxicity [38]. In this study, dietary chitosan could decrease the contents of C3 and IgM in juvenile GIFT. These results indicate that the nonspecific humoral immune response was suppressed. The effects of cadmium stress on tilapia were not eliminated. Similar results have been found in Japanese sea bass (*Lateolabrax japonicus*) fed diets with chitosan, where the C3 and IgM were decreased. The observed outcome could potentially be attributed to the phenomenon of “immune exhaustion” because of prolonged chitosan administration [39]. When the fish was exposed to environmental stress, it triggered activation of inflammation and immune response [40]. This study demonstrated that dietary chitosan significantly suppressed the expression of pro-inflammatory interleukin genes compared to the control group, indicating a lack of immune activation in fish. This observation may also account for the reduced serum C3 and IgM contents. Contrary to our findings, Chen et al. observed a significant increase in serum C3 and IgM content in gibel carp (Carassius auratus gibelio) with dietary chitosan supplementation [40]. However, inconsistent findings could be attributed to variations in fish species, feeding environments, test periods, and other factors. The reason for the decreasing C3 and IgM is unknown to us. Further study is required to explore the underlying mechanism behind this situation.

Aquatic organisms will trigger their antioxidant defense mechanism to eradicate an overabundance of reactive oxygen species (ROS) and free radicals once the oxidative stress within their bodies surpasses a specific threshold. This process effectively reduces the overall oxidative stress within the body, thereby preventing fish from experiencing damage and pathological changes caused by oxidation [41]. The superoxide dismutase (SOD), catalase (CAT), glutathione peroxidase (GSH-Px), and glutathione S-transferase (GST) constitute the primary antioxidant defense system in fish, playing a pivotal role in the elimination of free radicals. These enzymes serve as crucial indicators for assessing the health of the fish’s antioxidant system [42]. In this study, dietary chitosan could positively affect the values of CAT, SOD, GST, GSH-Px, total antioxidant capacity (T-AOC), and MDA and the gene expression of *cat*, *sod*, *gst*, and *gsh-px* in juvenile GIFT. This may be because chitosan could activate the antioxidant and immune functions of fish, promote the repair of damaged organs and tissues, and enhance the stress resistance of fish [43]. Chitosan and its derivatives could regulate the activity of antioxidant enzymes in fish, remove free radicals, and up-regulate the activities of SOD, T-AOC, and CAT [44]. According to the study conducted by Thilagar et al., the inclusion of chitosan in the diet had been found to significantly enhance the antioxidant activity of tilapia when exposed to lead stress, leading to a significant up-regulation in the activities of SOD, T-AOC, and CAT [45]. 

When fish experience stress, a notable quantity of superoxide free radicals is produced, leading to the peroxidation of lipid membranes and the generation of malondialdehyde (MDA). The excessive accumulation of MDA can potentially induce the formation of cross-linked polymers in proteins, nucleic acids, and other vital macromolecules for fish. Consequently, this may cause modifications in both the structure and functionality of cellular membranes. Therefore, the content of MDA can directly indicate the level of peroxidation in plasma membranes [46]. The molecular composition of chitosan, which contains numerous hydroxyl and amino groups, could eliminate reactive oxygen species. This results in a decrease in the buildup of MDA content within fish [47]. Mehrpak et al. explored the protective effect of chitosan on cadmium-induced oxidative stress in the liver of carp (*Cyprinuscarpio*) and they found that the addition of chitosan could effectively reduce the content of MDA in the liver of the carp in the cadmium-induced group [48].

In this study, we found that the trend of antioxidant enzyme activity in the liver and gills was inconsistent, indicating that the effects of chitosan and cadmium were different in tissue. The order of cadmium accumulation in the fish was found to be liver > gills [49]. Following chitosan feeding, there was a reduction in cadmium toxicity observed specifically in the gills, leading to a decrease in certain antioxidant parameters. In similar studies, Salaah et al. investigated the potential effects of chitosan on natural immunotoxicity and oxidative stress in lead-induced tilapia and they found that feeding lead-exposed tilapia with chitosan resulted in an increase in antioxidant enzyme activity in the liver, while it decreased in the gills [50]. Rahman et al. investigated the cadmium accumulation in tissues and organs of tilapia induced by cadmium exposure, and they found that the order of Cd^2+^ accumulation in tissues and organs was liver > gills [51].

One of the main osmoregulatory strategies in fish is to possess monocytes on the gill for transporting ions so that losses and gains due to osmotic challenges can be counteracted. In the gills, Na^+^-K^+^-ATPase and Ca^2+^-ATPase serve as the primary driving forces for ion transport [52]. Cd^2+^ binding to the thiol groups of cysteine residues located in the active centers of Na^+^-K^+^-ATPase and Ca^2+^-ATPase, thereby inhibiting enzyme activity. Consequently, this inhibition disrupted ion balance in osmoregulatory organs [53]. In this study, dietary chitosan could enhance the activities of Na^+^-K^+^-ATPase and Ca^2+^-ATPase in the gills of juvenile GIFT. These results suggest that chitosan could alleviate the effects of cadmium stress on Na^+^-K^+^-ATPase and Ca^2+^-ATPase activities. A similar study has shown that Raymond W.M. Kwong et al. discovered that the presence of cadmium ions hindered the activity of basolateral Ca^2+^-ATPase while investigating the oxidative stress induced by cadmium exposure in various tissues of rainbow trout (*Oncorhynchus mykiss*) [54]. Peles et al. observed that golden shiners (*Notemigonus crysoleucas*) experienced a shock response when exposed to the four highest concentrations of Cd (500, 800, 1100, and 1400 μg/L) for a duration of 24 h. During this exposure period, there was a significant decrease in both metabolic rate and Na^+^-K^+^-ATPase activity in the gills of the fish compared to those in the control group [55]. In this study, dietary chitosan could enhance the activities of Na^+^-K^+^-ATPase and Ca^2+^-ATPase, which reflected the repair and restoration of various energy requirement processes in the gills. The present study demonstrated that chitosan exhibited the ability to stimulate and augment the activity of antioxidant enzymes and ATPase in both the liver and gill tissues of juvenile tilapia, thereby effectively eliminating excessive reactive oxygen species within the body and maintaining cellular osmotic balance. Notably, the activation of specific enzymes displayed tissue- and organ-specificity, as well as temporal specificity, with enzyme activity being inhibited upon reaching the body’s tolerance threshold. However, the mechanism of chitosan is still unclear and needs further study.

Inflammatory responses can occur in fish under environmental stress and the production of inflammatory responses is usually associated with cytokines such as interferon γ (INF-γ), tumor necrosis factor α (TNF-α), transforming growth factor β (TGF-β), and interleukin (IL) [56]. INF-γ is a potent antiviral and immunomodulatory cytokine with a broad range of effects. Chitosan could stimulate the secretion of INF-γ, thereby inducing the activation of macrophages, NK cells, and T cells to synergistically control infection [57]. Additionally, chitosan could also promote the activation and proliferation of TGF-β, collectively regulating the differentiation and proliferation of various stress-resistant cell types while facilitating fibroblast division [58]. IL plays a crucial role as a member of the cytokines in the regulation of inflammatory response. In the presence of positively charged natural polymers, IL facilitates immune cell connectivity and coordination, thereby exerting its pivotal function in immune responses [59].

In this study, dietary chitosan could positively affect the gene expression of *tnf-α*, inf-*γ*, and *tgf-β* in the spleen, gills, head kidney, and liver of juvenile GIFT. This may be because, first, the positive feedback regulation between apoptosis and cadmium stress concentration led to an enhanced inflammatory response [60]. Additionally, cadmium stress triggered the proliferation and activation of T lymphocytes and activated the nuclear factor-kappa B (NF-κΒ) pathway to induce an inflammatory response, resulting in apoptosis and tissue necrosis in fish [61]. Choudhury et al. discovered that a 7-day exposure of murrel fish (*Channa punctatus*, Bloch) to a sub-lethal amount of Cd resulted in significant changes in the hematological, histological, and ultrastructural characteristics observed in monocytes/macrophages. The presence of inflammation was further confirmed through the increased expression of proinflammatory cytokines such as *tnf-α*, *il-1β*, *il-6*, and *il-12*. Moreover, both mRNA and protein levels of the anti-inflammatory cytokine *il-10* exhibited a decrease [62]. Second, chitosan could decrease the NF-κΒ pathway and thereby attenuate the inflammatory response [63]. Zheng et al. discovered that the addition of chitosan to the diet resulted in a decrease in the activity and expression level of both the gene and protein associated with inducible nitric oxide synthase. Additionally, there was a linear reduction in IL-1 content and gene expression. These findings suggest that incorporating chitosan into the diet may enhance antioxidant function while reducing pro-inflammatory mediators, potentially through its ability to inhibit the NF-κΒ pathway [64]. Third, the gene expression of *inf-γ* and *tgf-β* in the liver, gills, head kidney, and spleen of juvenile GIFT exhibited distinct patterns following the addition of dietary chitosan. This may be because Cd accumulation initially occurred in the gills [60]. Subsequently, Cd was transported to other fish organs like the liver and kidney via the circulatory system [65]. Therefore, there existed a slight variation in the binding affinity between chitosan and cadmium ions at different locations.

## 5. Conclusions

In conclusion, dietary chitosan could positively affect the growth performance, serum biochemical indices, antioxidant capacity, immune response, inflammatory response, and related gene expression in juvenile GIFT under cadmium stress. According to the analysis of second-order polynomial regression, it was found that the optimal dietary chitosan levels in juvenile GIFT was approximately 1.42% to 1.45%, based on its impact on Wf, WGR, SGR, and DGI.

## Figures and Tables

**Table 1:** Composition of the experimental diets for juvenile GIFT (g/kg of dried feed).

Ingredients	Chitosan Levels (%)				
	0	0.5	1.0	1.5	2.0
Chitosan	0.00	5.00	10.00	15.00	20.00
Soybean oil	20.00	20.00	20.00	20.00	20.00
Fish oil	20.00	20.00	20.00	20.00	20.00
Fish meal	80.00	80.00	80.00	80.00	80.00
Rapeseed meal	220.00	220.00	220.00	220.00	220.00
Soybean meal	330.00	330.00	330.00	330.00	330.00
Dextrin	243.90	238.90	233.90	228.90	223.90
Gelatin	50.00	50.00	50.00	50.00	50.00
Vitamin mixture ^1^	10.00	10.00	10.00	10.00	10.00
Mineral mixture ^2^	10.00	10.00	10.00	10.00	10.00
Choline chloride	5.00	5.00	5.00	5.00	5.00
Sodium chloride	5.00	5.00	5.00	5.00	5.00
Adhesive ^3^	5.00	5.00	5.00	5.00	5.00
Attractant ^4^	0.10	0.10	0.10	0.10	0.10
Preservative ^5^	1.00	1.00	1.00	1.00	1.00
Proximate composition (%)					
Crude protein	33.88	33.88	33.88	33.88	33.88
Crude fat	7.35	7.35	7.35	7.35	7.35
Ash	6.96	6.96	6.96	6.96	6.96
Moisture	9.36	9.36	9.36	9.36	9.36
Crude fiber	5.56	5.56	5.56	5.56	5.56
Nitrogen-free extract	36.89	36.89	36.89	36.89	36.89
Gross energy (Mcal/kg)	3.83	3.83	3.83	3.83	3.83

Note: ^1^ Vitamin mixture content per kilogram of dried feed: vitamin A 2500 IU; vitamin D_3_ 1200 IU; vitamin K_3_ 60 IU; folic acid 5 mg; vitamin B_1_ 10 mg; vitamin B_2_ 10 mg; vitamin B_6_ 20 mg; vitamin B_12_ 0.15 mg; niacin 40 mg; calcium pantothenate 20 mg; inositol 150 mg; biotin 0.2 mg; vitamin C 150 mg; vitamin E 60 mg. ^2^ Mineral mixture content per kilogram of dried feed: iron 15 mg; zinc 20 mg; manganese 2 mg; copper 1 mg; iodine 0.2 mg; selenium 0.05 mg; cobalt 0.25 mg; magnesium 0.06 mg; potassium 40 mg. ^3^ Adhesive: α-starch. ^4^ Attractants: nucleotides, betaine, amino acids, and taurine. ^5^ Preservative: sodium benzoate.

**Table 2:** Primers of test genes for juvenile GIFT.

Gene	Primer Sequence (5′→3′)	Amplicon Size (bp)	Gene Bank
*β-actin* ^1^	F: TGACCCAGATCATGTTTGAGACC	146	XM_031811226.1
	R: CTCGTAGATGGGTACTGTGTGGG		
*cat* ^2^	F: TGAATGAGGAGGAGCGACAGAGAC	118	XM_003447521.5
	R: CCATAGTCTGGATGCACAGCCTTC		
*gsh-px* ^3^	F: AACTTCCATTCCCCTGCGATGATG	123	NM_001279711.1
	R: CGTCAGGACCAACCAGGAACTTC		
*gst* ^4^	F: CACCCCAGATCCCAAACCCAAAC	140	XM_025897213.1
	R: CAACAAGCAGCACATCAGCAAGG		
*sod* ^5^	F: TCCAGCCTGCCCTCAAGTTTAATG	121	XM_003449940.5
	R: TCCCGTTTGATTGCCTCCATTAGC		
*ifn-γ* ^6^	F: ATGGGTGGTGTTTTGGAGTCGTATG	142	NM_001287402.1
	R: CTGAGTTGTTGGTGCTGCTGGAG		
*il-6* ^7^	F: CCGTCAAACATCGGCACTCT	99	XM_019350387.2
	R: CTCCTCCTCACTGCTGGTCA		
*il-8* ^8^	F: CTGTGAAGGCATGGGTGTGGAG	101	NM_001279704.1
	R: CGCAGTGGGAGTTGGGAAGAATC		
*il-10* ^9^	F: CCGTCAGGCTCAAGAAGCTC	81	XM_013269189.3
	R: GCGCTGAGTCTAAGTCGTCG		
*tnf-α* ^10^	F: GGAAGCAGCTCCACTCTGATGA	137	JF957373.1
	R: CACAGCGTGTCTCCTTCGTTCA		
*tgf-β* ^11^	F: CTCCTCCGACTTCCCTTTCAATGC	80	NM_001311325.1
	R: TGCCTCCTCTCCACTGAGTGATTC		

Note: F: Forward primer; R: Reverse primer; ^1^ *β-actin:* Internal reference gene; ^2^ *cat*: Catalase; ^3^ *gsh-px*: Glutathione peroxidase; ^4^ *gst*: Glutathione S-transferase; ^5^ *sod*: Superoxide dismutase; ^6^ *inf-γ*: interferon γ; ^7^ *il-6*: Interleukin 6; ^8^ *il-8*: Interleukin 8; ^9^ *il-10*: Interleukin 10; ^10^ *tnf-α*: Tumor necrosis factor α; ^11^ *tgf-β*: Transforming growth factor β.

**Table 3:** Effects of dietary chitosan on growth performance of juvenile GIFT under cadmium stress.

Index	Chitosan Levels (%)					F-Value	*p*-Value
	0	0.5	1.0	1.5	2.0		
SR ^1^ (%)	98.89 ± 1.11 ^b^	99.76 ± 0.11 ^b^	100 ± 0 ^a^	100 ± 0 ^a^	100 ± 0 ^a^	3.416	0.008
Wi ^2^ (g)	21.36 ± 0.24	21.36 ± 0.24	21.36 ± 0.24	21.36 ± 0.24	21.36 ± 0.24	/	/
Wf ^3^ (g)	56.07 ± 3.01 ^b^	71.99 ± 1.87 ^a^	78.89 ± 1.46 ^a^	73.85 ± 3.06 ^a^	77.99 ± 5.56 ^a^	0.818	0.003
WGR ^4^ (%)	162.52 ± 14.11 ^b^	237.06 ± 8.75 ^a^	269.38 ± 6.83 ^a^	245.78 ± 14.34 ^a^	265.18 ± 26.02 ^a^	100.704	0.004
SGR ^5^ (%/d)	1.6 ± 0.09 ^b^	2.03 ± 0.04 ^a^	2.18 ± 0.03 ^a^	2.06 ± 0.07 ^a^	2.15 ± 0.12 ^a^	3.383	0.003
DGI ^6^ (%/d)	5.43 ± 0.16 ^b^	6.16 ± 0.07 ^a^	6.43 ± 0.05 ^a^	6.23 ± 0.12 ^a^	6.39 ± 0.21 ^a^	3.473	0.002
CF ^7^ (%)	2.11 ± 0.28 ^b^	3.74 ± 0.13 ^a^	3.62 ± 0.11 ^a^	3.58 ± 0.15 ^a^	3.47 ± 0.16 ^a^	15.216	<0.001
HSI ^8^ (%)	1.69 ± 0.45	0.98 ± 0.13	1.09 ± 0.12	1.37 ± 0.13	1.61 ± 0.09	3.733	0.185
VSI ^9^ (%)	12.5 ± 0.59 ^ab^	13.41 ± 0.87 ^a^	11.66 ± 0.47 ^ab^	10.78 ± 0.77 ^b^	12.93 ± 0.12 ^a^	5.960	0.084
SI ^10^ (%)	0.51 ± 0.17	0.55 ± 0.02	0.46 ± 0.10	0.44 ± 0.04	0.39 ± 0.04	2.086	0.760
FCR ^11^	1.7 ± 0.02 ^a^	1.63 ± 0.01 ^b^	1.59 ± 0.02 ^b^	1.53 ± 0.03 ^c^	1.53 ± 0.02 ^c^	92.351	<0.001

Notes: All above data are mean ± SE (*n* = 3). Different superscript letters in the same row indicate significant differences among the data (*p* < 0.05). ^1^ SR: Survival rate; ^2^ Wi: Initial weight; ^3^ Wf: Final weight; ^4^ WGR: Weight gain rate; ^5^ SGR: Specific growth rate; ^6^ DGI: Daily growth index; ^7^ CF: Condition factor; ^8^ VSI: Viscera index; ^9^ HSI: Hepatosomatic index; ^10^ SI: Spleen index; ^11^ FCR: Feed conversion ratio.

**Table 4:** Effects of dietary chitosan on serum biochemical indexes of juvenile GIFT under cadmium stress.

Index	Chitosan Levels (%)					F-Value	*p*-Value
	0	0.5	1.0	1.5	2.0		
CHE ^1^ (UI/L)	6130.73 ± 141.94 ^d^	6734.67 ± 189.41 ^c^	6749.57 ± 187.57 ^b^	6981.88 ± 121.74 ^bc^	7306.87 ± 136.77 ^a^	14.483	<0.001
ALB ^2^ (g/L)	7.63 ± 0.96 ^c^	8.52 ± 0.69 ^ab^	8.41 ± 0.74 ^b^	8.59 ± 0.62 ^ab^	9.07 ± 0.75 ^a^	8.707	0.003
GPT ^3^ (U/mL)	0.28 ± 0.03 ^a^	0.24 ± 0.05 ^b^	0.21 ± 0.05 ^c^	0.19 ± 0.04 ^d^	0.18 ± 0.02 ^d^	26.617	<0.001
GOT ^4^ (U/mL)	0.13 ± 0.02 ^a^	0.11 ± 0.01 ^b^	0.11 ± 0.01 ^b^	0.10 ± 0.01 ^b^	0.09 ± 0.01 ^c^	16.991	<0.001
LDH ^5^ (U/L)	307.79 ± 8.77 ^c^	346.81 ± 24.31 ^b^	352.4 ± 15.96 ^b^	391.27 ± 14.96 ^a^	388.93 ± 16.26 ^a^	17.562	<0.001
ALP ^6^ (King unit/100 mL)	8.42 ± 0.44 ^d^	8.82 ± 0.62 ^c^	9.09 ± 0.36 ^b^	9.26 ± 0.55 ^a^	9.45 ± 0.89 ^a^	26.991	<0.001
ACP ^7^ (King unit/100 mL)	93.29 ± 3.75 ^c^	121.94 ± 9.11 ^b^	153.91 ± 9.68 ^a^	161.41 ± 9.52 ^a^	156.39 ± 8.51 ^a^	16.956	<0.001
IgM ^8^ (μg/mL)	2967.55 ± 30.70 ^a^	2926.3 ± 21.72 ^a^	2831.95 ± 37.43 ^b^	2776.86 ± 36.79 ^b^	2653.36 ± 21.06 ^c^	43.167	<0.001
C3 ^9^ (μg/mL)	2.52 ± 0.23 ^a^	2.04 ± 0.13 ^b^	1.74 ± 0.23 ^bc^	1.84 ± 0.19 ^bc^	1.64 ± 0.20 ^c^	12.375	<0.001
LZM ^10^ (μg/mL)	96.35 ± 1.22 ^c^	143.76 ± 12.66 ^b^	159.91 ± 10.33 ^ab^	176.75 ± 3.23 ^a^	179.61 ± 7.10 ^a^	17.461	<0.001

Notes: All above data are mean ± SE (*n* = 3). Different superscript letters in the same row indicate significant differences among the data (*p* < 0.05). ^1^ CHE: Cholinesterase; ^2^ ALB: Albumin; ^3^ GPT: Glutamic pyruvic transaminase; ^4^ GOT: Glutamic oxaloacetic transaminase; ^5^ LDH: Lactate dehydrogenase; ^6^ ALP: Alkaline phosphatase; ^7^ ACP: Acid phosphatase; ^8^ IgM: Immunoglobulin M; ^9^ C3: Complement 3; ^10^ LZM: Lysozyme.

**Table 5:** Effects of dietary chitosan on antioxidant enzyme activity in the serum of juvenile tilapia under cadmium stress.

Index	Chitosan Levels (%)					F-Value	*p*-Value
	0	0.5	1.0	1.5	2.0		
SOD ^1^ (U/mL)	12.04 ± 0.03 ^d^	18.20 ± 0.66 ^c^	19.56 ± 0.65 ^c^	24.48 ± 0.65 ^a^	22.31 ± 0.10 ^b^	86.726	<0.001
CAT ^2^ (U/mL)	6.44 ± 0.51 ^d^	8.45 ± 0.26 ^c^	10.29 ± 0.47 ^b^	11.99 ± 0.36 ^a^	12.67 ± 0.89 ^a^	27.147	<0.001
GST ^3^ (U/mL)	9.78 ± 0.16 ^a^	8.42 ± 0.17 ^b^	8.69 ± 0.27 ^b^	8.48 ± 0.17 ^b^	7.65 ± 0.20 ^c^	14.677	<0.001
GSH-Px ^4^ (U/mL)	23.45 ± 0.42 ^a^	21.89 ± 0.14 ^b^	21.37 ± 0.04 ^b^	20.45 ± 0.26 ^c^	19.73 ± 0.38 ^c^	25.276	<0.001
T-AOC ^5^ (mmol/L)	0.23 ± 0.01 ^a^	0.22 ± 0.01 ^a^	0.20 ± 0.01 ^b^	0.20 ± 0.01 ^bc^	0.19 ± 0.01 ^c^	14.863	<0.001
MDA ^6^ (nmol/mL)	3.80 ± 0.34 ^a^	3.41 ± 0.14 ^b^	3.39 ± 0.12 ^b^	3.28 ± 0.14 ^b^	3.00 ± 0.09 ^c^	9.497	<0.001

Notes: All above data are mean ± SE (*n* = 3). Different superscript letters in the same row indicate significant differences among the data (*p* < 0.05). ^1^ SOD: Superoxide dismutase; ^2^ CAT: Catalase; ^3^ GST: Glutathione S-transferase; ^4^ GSH-Px: Glutathione peroxidase; ^5^ T-AOC: Total antioxidant capacity; ^6^ MDA: Malondialdehyde.

**Table 6:** Effects of dietary chitosan on antioxidant enzyme activity in the liver of juvenile GIFT under cadmium stress.

Index	Chitosan Levels (%)					F-Value	*p*-Value
	0	0.5	1.0	1.5	2.0		
SOD ^1^ (U/mgprot)	84.54 ± 5.74 ^b^	95.00 ± 3.72 ^b^	117.83 ± 7.19 ^a^	119.26 ± 9.06 ^a^	124.03 ± 10.78 ^a^	14.243	<0.001
CAT ^2^ (U/mgprot)	37.71 ± 4.48 ^c^	55.21 ± 3.95 ^b^	56.96 ± 1.33 ^b^	68.13 ± 2.25 ^a^	59.23 ± 4.76 ^b^	16.054	<0.001
GST ^3^ (U/mgprot)	86.15 ± 5.78 ^c^	98.39 ± 6.46 ^bc^	115.21 ± 4.3 ^b^	152.36 ± 7.66 ^a^	152.85 ± 11.69 ^a^	28.495	<0.001
GSH-Px ^4^ (U/mgprot)	0.36 ± 0.02 ^b^	0.46 ± 0.02 ^a^	0.43 ± 0.01 ^a^	0.44 ± 0.02 ^a^	0.44 ± 0.03 ^a^	4.385	<0.001
T-AOC ^5^ (mmol/L)	80.29 ± 1.36 ^c^	95.54 ± 5.85 ^b^	93.62 ± 5.2 ^b^	103.08 ± 7.92 ^a^	105.45 ± 10.86 ^a^	20.775	<0.001
MDA ^6^ (nmol/mgprot)	1.47 ± 0.37 ^a^	0.7 ± 0.02 ^b^	0.61 ± 0.01 ^c^	0.51 ± 0.01 ^d^	0.43 ± 0.02 ^e^	923.167	<0.001

Notes: All above data are mean ± SE (*n* = 3). Different superscript letters in the same row indicate significant differences among the data (*p* < 0.05). ^1^ SOD: Superoxide dismutase; ^2^ CAT: Catalase; ^3^ GST: Glutathione S-transferase; ^4^ GSH-Px: Glutathione peroxidase; ^5^ T-AOC: Total antioxidant capacity; ^6^ MDA: Malondialdehyde.

**Table 7:** Effects of dietary chitosan on antioxidant enzyme activity in the gills of juvenile GIFT under cadmium stress.

Index	Chitosan Levels (%)					F-Value	*p*-Value
	0	0.5	1.0	1.5	2.0		
SOD ^1^ (U/mgprot)	124.12 ± 10.8 ^d^	135.27 ± 12.26 ^c^	158.47 ± 15.08 ^b^	171.91 ± 16.02 ^a^	158.40 ± 13.24 ^b^	23.888	<0.001
CAT ^2^ (U/mgprot)	28.53 ± 1.19 ^a^	27.29 ± 2.49 ^b^	26.87 ± 2.40 ^b^	27.09 ± 1.40 ^b^	26.34 ± 2.20 ^b^	5.259	<0.001
GST ^3^ (U/mgprot)	415.7 ± 12.81 ^a^	359.55 ± 19.45 ^b^	353.16 ± 24.53 ^b^	262.57 ± 11.17 ^c^	243.84 ± 13.11 ^c^	38.039	<0.001
GSH-Px ^4^ (U/mgprot)	5.72 ± 0.02 ^c^	7.47 ± 0.22 ^b^	9.81 ± 0.16 ^a^	10.00 ± 0.40 ^a^	9.56 ± 0.19 ^a^	63.862	<0.001
T-AOC ^5^ (mmol/L)	0.48 ± 0.02 ^b^	0.52 ± 0.01 ^a^	0.53 ± 0.02 ^a^	0.55 ± 0.01 ^a^	0.56 ± 0.02 ^a^	8.272	0.003
MDA ^6^ (nmol/mgprot)	3.4 ± 0.19 ^a^	2.33 ± 0.34 ^b^	1.75 ± 0.15 ^bc^	1.88 ± 0.22 ^bc^	1.39 ± 0.29 ^c^	15.753	<0.001

Notes: All above data are mean ± SE (*n* = 3). Different superscript letters in the same row indicate significant differences among the data (*p* < 0.05). ^1^ SOD: Superoxide dismutase; ^2^ CAT: Catalase; ^3^ GST: Glutathione S-transferase; ^4^ GSH-Px: Glutathione peroxidase; ^5^ T-AOC: Total antioxidant capacity; ^6^ MDA: Malondialdehyde.

## Data Availability

The data that support the findings of this study are available from the corresponding author(s) upon reasonable request.
